# Mitochondrial genomes of the Baltic clam *Macoma balthica* (Bivalvia: Tellinidae): setting the stage for studying mito-nuclear incompatibilities

**DOI:** 10.1186/s12862-014-0259-z

**Published:** 2014-12-21

**Authors:** Alice Saunier, Pascale Garcia, Vanessa Becquet, Nathalie Marsaud, Frédéric Escudié, Eric Pante

**Affiliations:** Littoral, Environnement et Sociétés, UMR 7266 CNRS, Université de La Rochelle, 2 rue Olympe de Gouges, La Rochelle, 17000 France; GeT-PlaGe, Genotoul, INRA Auzeville, Castanet-Tolosan, 31326 France

**Keywords:** NADH dehydrogenase, ATP synthase, Next-generation sequencing, OXPHO chain, Positive selection, Inter-specific hybridization, Hybrid zone

## Abstract

**Background:**

Allopatric divergence across lineages can lead to post-zygotic reproductive isolation upon secondary contact and disrupt coevolution between mitochondrial and nuclear genomes, promoting emergence of genetic incompatibilities. A previous *F*_ST_ scan on the transcriptome of the Baltic clam *Macoma balthica* highlighted several genes potentially involved in mito-nuclear incompatibilities (MNIs). As proteins involved in the mitochondrial oxidative phosphorylation (OXPHO) chain are prone to MNIs and can contribute to the maintenance of genetic barriers, the mitochondrial genomes of six *Ma. balthica* individuals spanning two secondary contact zones were sequenced using the Illumina MiSeq plateform.

**Results:**

The mitogenome has an approximate length of 16,806 bp and encodes 13 protein-coding genes, 2 rRNAs and 22 tRNAs, all located on the same strand. *atp8*, a gene long reported as rare in bivalves, was detected. It encodes 42 amino acids and is putatively expressed and functional. A large unassigned region was identified between *rrnS* and *tRNA*^*Met*^ and could likely correspond to the Control Region. Replacement and synonymous mutations were mapped on the inferred secondary structure of all protein-coding genes of the OXPHO chain. The *atp6* and *atp8* genes were characterized by background levels of replacement mutations, relative to synonymous mutations. However, most *nad* genes (notably *nad2* and *nad5*) were characterized by an elevated proportion of replacement mutations.

**Conclusions:**

Six nearly complete mitochondrial genomes were successfully assembled and annotated, providing the necessary roadmap to study MNIs at OXPHO loci. Few replacement mutations were mapped on mitochondrial-encoded ATP synthase subunits, which is in contrast with previous data on nuclear-encoded subunits. Conversely, the high population divergence and the prevalence of non-synonymous mutations at *nad* genes are congruent with previous observations from the nuclear transcriptome. This further suggest that MNIs between subunits of Complex I of the OXPHO chain, coding for NADH dehydrogenase, may play a role in maintaining barriers to gene flow in *Ma. balthica*.

**Electronic supplementary material:**

The online version of this article (doi:10.1186/s12862-014-0259-z) contains supplementary material, which is available to authorized users.

## Background

The mitochondrial electron transport chain, a central component of cellular energy production, relies on the coevolution of mitochondrial- and nuclear-encoded genes to function [1,2]. Mito-nuclear coevolution is however easily disrupted because genomes have different mutation rates, modes of inheritance, number and speed of recombination events, effective population size and selection pressures [3-5]. Such disruption can cause the emergence and maintenance of mito-nuclear incompatibilities (MNIs, among other types of genomic incompatibilities [6-9]) that are described by the Dobzhansky-Muller model [10,11].

Dobzhansky-Muller incompatibilities have been implicated in intrinsic post-zygotic barriers [8] and involved in mito-nuclear gene interactions in the fruit fly *Drosophila* [5], the parasitoid wasp *Nasonia* [12-14], the Atlantic eel *Anguilla* [15,16] and the marine copepod *Trigriopus californicus* [4,17,18]. Studying reproductive isolation is especially relevant in the marine environment, where many marine organisms have a high fecundity, large population sizes and a high dispersive potential [19], and where physical barriers to dispersal are not readily apparent. Endogenous barriers to gene flow may therefore be an important factor structuring marine populations in this environment. In addition, marine taxa can be sensitive to glaciation cycles that can promote the establishment of secondary contact zones and therefore lead to admixture of genetically divergent backgrounds [20].

In this context, the Baltic clam *Macoma balthica* (Mollusca: Bivalvia: Tellinidae) is a noteworthy model for studying the role of MNIs in structuring marine populations. This infaunal tellinid bivalve occurs on sandy-mud flats from the upper intertidal to the subtidal, in parts of the northern hemisphere. Its natural European range extends from eastern Russia (Pechora Sea) to southern France (Gironde Estuary). *Ma. balthica* is characterized by a complex colonization history in Europe. With the opening of the Bering Strait, several trans-arctic invasion events of Pacific populations into the Atlantic have occurred, leading to secondary contact of divergent lineages in the Bay of Biscay [21] and in the Baltic and White seas [22], allowing gene flow between previously-isolated populations. The long periods of reproductive isolation between these populations (~0.1-1.8 My between Bay of Biscay populations [23]; ~ 2–3.5 My between *Ma. balthica balthica* and *Ma. balthica rubra* subspecies [22]) represent strong potential for the accumulation of genetic incompatibilities.

A recent *F*_ST_ scan based on transcriptome data highlighted outlier loci coding for nuclear subunits of the F_O_F_1_-ATP synthase complex (subunits alpha, gamma and O) and a putative isoform of the NADH deshydrogenase [24], which are involved in the oxidative phosphorylation (OXPHO) mitochondrial chain [1,2]. These protein complexes are composed of subunits encoded by both nuclear and mitochondrial genes [2] and require tight mito-nuclear coevolution to be fully operational. They are therefore prime candidates for the establishment of MNIs following secondary contact between previously allopatric populations [6,17], which may constitute endogenous barriers to gene flow.

Sequences from only two mitochondrial genes have been published to date for this species (*cox3* [22]; *cox1* [21,23]). To work toward the diagnosis of MNIs in *Ma. balthica* and further test the hypothesis of mito-nuclear coevolution breakdown among *Ma. balthica* lineages, additional mitochondrial data are therefore required for comparison with the nuclear transcriptomic data at hand. Although several mitogenomes have been sequenced for marine bivalves (*e.g.* [25,26]) only one is currently available for the Tellinidae (*Moerella iridescens* [26]). However, mitochondrial sequences of *Mo. iridescens* are highly divergent from those of *Ma. balthica* (raw p-distance at *cox1* of 17%). We have therefore set to sequence the mitogenomes of six *Ma. balthica* individuals spanning two secondary contact zones (*i.e.* across the Kattegat strait and the Brittany peninsula) to test whether mitochondrial genes interacting with previously-detected nuclear outlier loci show stronger divergence and selection pressures, compared to other mitochondrial genes. Our intents were (i) to detect and map protein-coding genes (PCGs) involved in the OXPHO chain, (ii) to estimate the degree of divergence and selection pressures across lineages for all PCGs, and (iii) to map mutations onto predicted secondary structure of mitochondrial membrane-embedded protein to help detect potential incompatibilities among mitochondrial and nuclear OXPHO subunits. In particular, we characterize a putative *atp8* gene and discuss its functionality, as (i) it is absent in about half of the published bivalve mitogenomes, and (ii) its role is potentially relevant to the study of MNIs in *Ma. balthica*.

## Methods

### Sample collection and DNA extraction

Previous work on *Ma. balthica* suggested the presence of at least three divergent mitochondrial clades in Europe [21-23], which were targeted in our sampling: a Baltic lineage, and two Atlantic lineages that are separated by the French Finistère peninsula. *Ma. balthica* specimens were collected from 2003 to 2006 (A10, M12, W4, W20 and F17) and 2013 (A6) along European coasts, ranging from Aytré, France to Tvärminne, Finland (Table 1). Total genomic DNA was extracted from foot muscle tissue using the Dneasy™ Tissue Kit (Qiagen, Germany) following the manufacturer's protocol and stored at −20°C until further analyses.

**Table 1:** **Sampling sites and mitochondrial** ***cox1*** **haplotypes of the sequenced specimens of** ***Ma. balthica*** **.** ***cox1*** **haplotypes are described in Becquet** ***et al.*** [21] **and are noted H1 to H5. H1 and H2 are common of the Bay of Biscay, H3 of the English Channel, H4 and H5 of North and Baltic seas, respectively**

**Sampling site**	**Country**	**Code**	**Latitude**	**Longitude**	**GB Accession no.**	***cox1*** **haplotype**
Aytré	France	A	46.126	−1.1306	A10: KM373200	H1
Aytré	France	A	46.126	−1.1306	A6: KM373201	H2
Mont-Saint-Michel	France	M	48.438	−1.5153	M12: KM373202	H3
Balgzand	Netherlands	W	52.9301	+4.7953	W4: KM373203	H3
Balgzand	Netherlands	W	52.9301	+4.7953	W20: KM373204	H4
Tvärminne	Finland	F	59.4883	+21.2051	F17: KM373205	H5

Initially eight mitogenomes were sequenced, corresponding to two individuals per mitochondrial lineage, as inferred from 313 bp of *cox1* by Becquet et al. [21] (and hereon referred to as “*cox1* haplotypes”). Six nearly-complete mitogenomes were eventually obtained, as Long-Range PCR (detailed below) failed for two individuals. They correspond to *cox1* haplotypes H1 to H5 (Table 1). H1-H4 were originally described in [21] and H5 is a newly described haplotype.

### Primer design and Long-Range PCR amplification of mtDNA

Three pairs of primers were designed using Primer-BLAST [27] to amplify the mitochondrial genome of *Ma. balthica* (Additional files 1 and 2). The first two pairs were designed based on *cox1* and *cox3* sequences available on GenBank for *Ma. balthica* [22,23]. Primer length was maximized (28–31 bp) to enhance stability during Long-Range PCR (LR-PCR). When the two-step mitogenome amplification did not succeed, one additional primer set (Palumbi *et al.* 1996 in [28]) was used to prime within *rrnL*. These three primer pairs allowed the amplification of 3 to 16.5 kbp. LR-PCR products were visualised by electrophoresis on a 0.7% agarose gel stained with GelRed™ (Biotium, Hayward, CA, USA) to assess quality.

### Library preparation and DNA sequencing

The quantity of LR-PCR DNA templates (prior and during library preparation) was assessed by spectrophotometry (Nanodrop ND-800, Thermo Scientific, Waltham, MA, USA), fluorometry (Qubit, Invitrogen, Carlsbad, NM, USA) and quantitative PCR (PicoGreen dosage with Quant-iT™ PicoGreen® dsDNA Assay Kit, Invitrogen on ABI 7900HT, Applied Biosystems). After quality controls, six libraries (one per individual) were prepared using the TruSeq DNA Sample Prep Kits v2 and TruSeq Universal Adapters (Illumina, San Diego, CA, USA). A TruSeq Universal Adapter was used for each DNA library in order to separate reads from different individuals after DNA sequencing. Library sizes were checked on BioAnalyzer chips (Agilent Technologies, Santa Clara, CA, USA). Paired-end library sequencing was carried out on the Illumina MiSeq plateform (2 × 250 bp chemistry) at the GeT-PlaGe lab (GenoToul, Toulouse, France).

### Sequence cleaning, trimming, assembly and gene annotation

Genomes were assembled in two rounds, using different read quality filters and assembly parameters, to optimize contig length while controlling for quality. In the first round, reads were de-multiplexed and quality-filtered (length > 10 nucleotides; Q ≥ 28 on 50% of read length) using the FastX toolkit (http://hannonlab.cshl.edu/fastx_toolkit/). Duplicated sequences were removed and adapters were clipped. Sequences (Illumina 1.9 quality scores) were checked for quality before and after FastX filtering using FastQC v.0.10.1 [29]. Assembly was performed in Velvet v.1.2.09 [30], and parameters (coverage cut-off and k-mer size) were optimized using a custom R script to minimize the number of contigs and maximize contig lengths (total length and N50 parameter). Optimized k-mer sizes were 89 bp (W20), 97 bp (F17), 103 bp (A10, M12), 107 bp (A6) and 119 bp (W4). Optimized k-mer coverages were 250 (A6, A10, F17, M12, W4) and 300 (W20). Assemblies were checked in Tablet v.1.13.05.17 [31].

In the second round of assemblies, we used more stringent quality filters (length > 250 nucleotides; Q ≥ 29 on 90% of read length), and fixed k-mer length and k-mer coverage to 247 bp and 100, respectively. In the second assembly, contigs from the first round were used as a reference; Sanger sequences for parts of the *cox1* and *cox3* genes were included to fill the gaps flanking the LR-PCR primer sequences. Sanger sequencing was performed by GATC Biotech (Konstanz, Germany) using an ABI 3730xl automated DNA Analyzer (Perkin-Elmer Applied Biosystems, Foster City, CA, USA).

All contigs from the first and second assemblies and parts of *cox1*, *cox3* sequences were aligned using Sequencher™ v.5.0.1 (Gene Codes Corporation, Ann Arbor, MI, USA). Resulting contigs (1 for A6, A10, W4, F17 and 2 for M12, W20) were searched on GenBank (Tellinidae non-redundant protein sequence database) in order to control for contaminants. We also searched for the presence of these contigs among the 454 transcriptome sequences available for *Ma. balthica* [24] using a local BLAST database and tblastx (BLAST toolkit v. 2.2.25; [32]). Mitochondrial genome annotations were then performed on the MITOS WebServer, first using default parameters [33] and second using advanced parameters (BLAST E-value = 1×10^−4^, Start/Stop Range = 40 and Final Maximum Overlap = 10) in order to improve annotation quality and start/stop codon delimitation.

### Quality control and gene boundary delimitation

Mitochondrial genomes were aligned using Sequencher™ and each ambiguity and indel was individually checked by eye in Tablet. The gene boundaries automatically detected by MITOS were checked individually in Sequencher™ to correct for misalignments. The location and size of the 13 PCGs were evaluated by comparing the location of start and stop codons with that of published mitochondrial genomes (the tellinid *Mo. iridescens*: GenBank accession number JN398362.1 [26] and the venerid *Meretrix lusoria*: GQ903339.1 [25]). Congruence among the PCG hydrophobic profiles of these species was assessed using Unipro UGENE v1.12.0 [34]. The map of mitogenome of *Ma. balthica* was produced using GenomeVx [35].

### tRNA and ATP8 protein structure characterization

tRNA secondary structures were inferred using MITOS in default search mode [33]. GC content was calculated with UGENE. Predictions of transmembrane alpha-helices and hydrophilic helices for the ATP8 protein were inferred with TMHMM v.2.0 [36] and were compared with previously characterized ATP8 proteins from other bivalves. ATP8 amino-acid sequences were aligned with MUSCLE v.3.8.31 [37] with manual adjustments and a graphical representation was prepared using Texshade [38].

### Genetic divergence and phylogenetic analysis

Genetic divergence among four pairs of haplotypes of *Ma. balthica* individuals was analyzed using the *ape* v.3.0-8 [39] and *seqinr* v.3.0-7 [40] R packages [41]. The TN93 [42] model of nucleotide substitution was determined as the most likely for our dataset based on the BIC scores calculated in jModelTest 2 [43]. TN93 genetic distances within and between specimens with different *cox1* haplotypes were calculated within a 200 bp window sliding every 10 bp.

Nucleotide diversity for synonymous (π_S_) and non-synonymous (π_A_) substitutions was calculated across all PCGs for all mitogenomes (n = 6) and *Ma. balthica rubra* mitogenomes (n = 5), using a 200 bp window sliding every 10 bp (DnaSP v.5.10.01 [44]). Haplotype networks for each of the 13 PCGs were built using the *ape* and *pegas* v.0.4-2 [45] R packages. Finally, the secondary structure of PCGs was inferred with Protter v.1.0 [46]. Fisher’s exact tests (as implemented in R) were computed on each PCG to test for the random distribution of (i) all mutations and (ii) non-synonymous mutations across mitochondrial compartments (extra-, inter- and intra-membrane).

## Results

### Mitochondrial genome assembly and annotation

One Illumina MiSeq run produced 22.4 M raw reads, representing 3.02 Gbp after quality filtering (*i.e.* 4.78% reads deleted). GC content varied between 36 and 40% (average: 37%). The second round of genome assemblies yielded a median contig size (N50) of 6,893 bp and a maximum contig size averaging 7,449 bp.

### Mitochondrial genome organization, PCGs & codon usage

**Figure 1:**
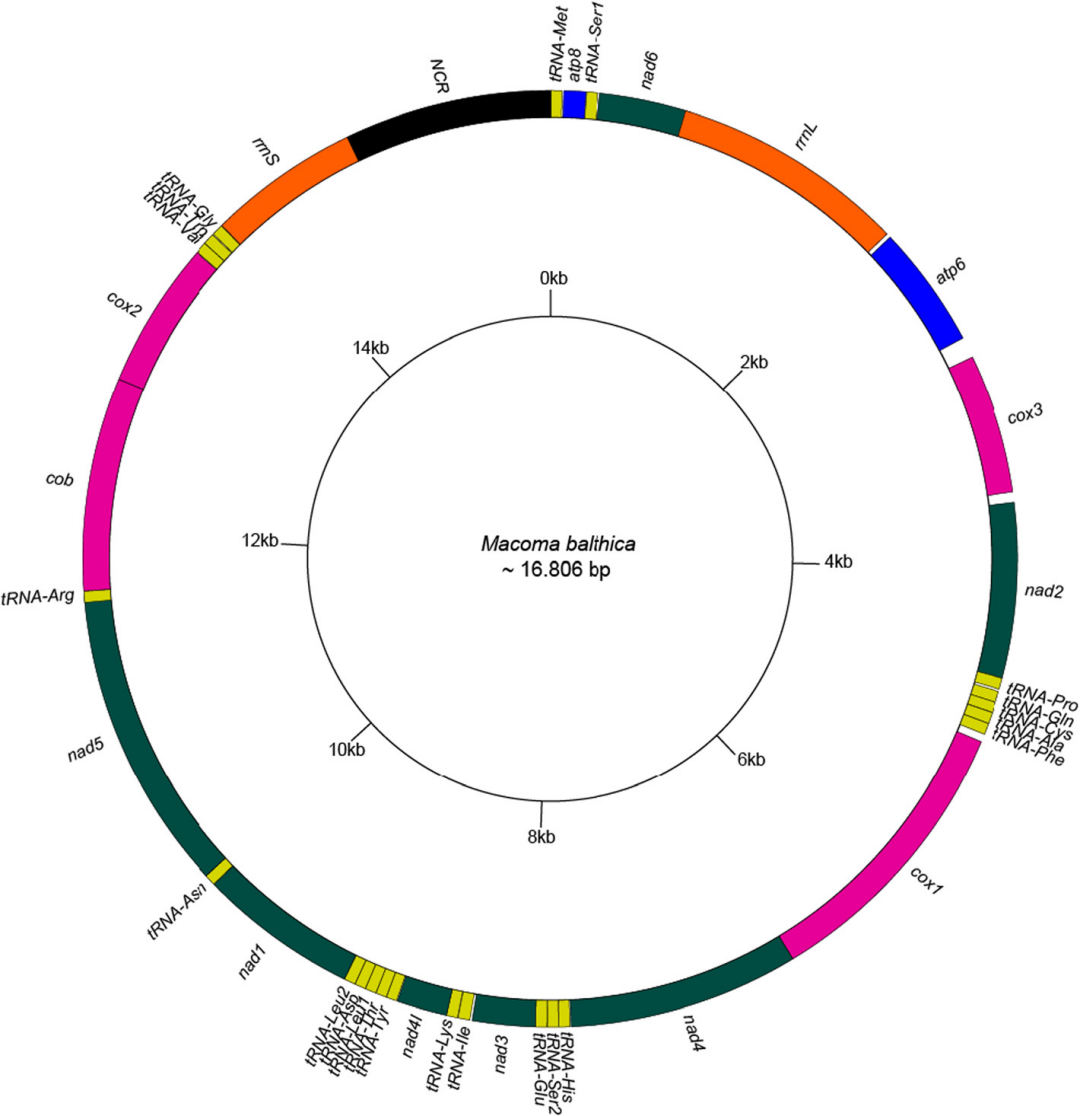
**Map of the mitochondrial genome of** ***Ma. balthica*** **, based on specimen A10.** NCR indicates the largest non-coding region.

Six nearly complete mitochondrial genomes from five mitochondrial lineages (as represented by the *cox1* haplotypes of Becquet et al. [21]) were obtained. The mitogenome of individual A10 from Aytré is considered as our reference genome hereon. The size of the mitogenome is estimated at 16,806 bp (A6, M12, W4: 16,805; W20: 16,807 and F17: 16,794 bp). It is composed of 37 genes, including 13 PCGs among which an *atp8* gene, two ribosomal RNA genes and 22 transfer RNA genes (Table 2 and Figure 1). All genes are encoded on the same strand, and gene arrangement is identical among the six mitogenomes sequenced.

**Table 2:** **Main features of the mitogenome of** ***Ma. balthica***

**Gene**	**Position**		**Size**		**Intergenic nucleotides** ^**1**^	**Codon** ^**2**^	
	**From**	**To**	**Nucleotides**	**Amino acids**		**Start**	**Stop**
*tRNA-Met*	1	65	65		9		
*atp8*	75	203	129	42	3	ATT	TAA
*tRNA-Ser1*	207	275	69		10		
*nad6*	286	819	534	177	−32	ATT	TAA
*rrnL*	788	2148	1361		25		
*atp6*	2174	2884	711	236	121	ATG (GTG - F17)	TAA
*cox3*	3006	3818	813	270	60	GTG	TAA
*nad2*	3879	4889	1011	336	0	ATA	TAA
*tRNA-Pro*	4890	4955	66		13		
*tRNA-Gln*	4969	5034	66		−1		
*tRNA-Cys*	5034	5094	61		1		
*tRNA-Ala*	5096	5161	66		0		
*tRNA-Phe*	5162	5227	66		46		
*cox1*	5274	6944	1671	556	0	ATA	TAA
*nad4*	6945	8285	1341	446	6	ATA	TAG
*tRNA-His*	8292	8356	65		1		
*tRNA-Ser2*	8358	8422	65		1		
*tRNA-Glu*	8424	8490	67		0		
*nad3*	8491	8853	363	120	13	TTG	TAG (TAA - W20)
*tRNA-Ile*	8867	8935	69		2		
*tRNA-Lys*	8938	9002	65		0		
*nad4l*	9003	9293	291	96	5	GTG	TAA
*tRNA-Tyr*	9299	9360	62		0		
*tRNA-Thr*	9361	9424	64		0		
*tRNA-Leu1*	9425	9489	65		0		
*tRNA-Asp*	9490	9553	64		1		
*tRNA-Leu2*	9555	9620	66		1		
*nad1*	9622	10545	924	307	1	ATG	TAG
*tRNA-Asn*	10547	10611	65		0		
*nad5*	10612	12405	1794	597	−50	TTG	TAA
*tRNA-Arg*	12356	12420	65		1		
*cob*	12422	13660	1239	412	1	ATT	TAA
*cox2*	13662	14516	855	284	3	ATG	TAA
*tRNA-Val*	14520	14586	67		0		
*tRNA-Trp*	14587	14652	66		5		
*tRNA-Gly*	14658	14723	66		3		
*rrnS*	14727	15606	880		1200*		

Main features of the mitogenome of Ma. balthica, based on specimen A10.

^1^Nucleotide positions are indicated by the numbers separating the different mitochondrial genes. Overlapping nucleotides between adjacent genes are noticed by negative numbers.

^2^ *atp6* and *nad3* genes presented specific codon for one mitogenome. Codon is indicated in brackets following by mtDNA reference.

*See text.

In total, PCGs encode 3,879 amino acids (aa). With a size of 1,794 bp (597 aa), *nad5* is the largest PCGs and *cox1*, *nad4*, *cob* and *nad2* genes exceed 1,000 bp. Conversely, *nad3* and *nad4l* genes are smaller than 400 bp lengths and *atp8* gene is the smallest gene with 129 bp (42 aa).

Five different initiation codons were identified. Three PCGs begin with ATT (*atp8, nad6, cob*), three with ATG (*atp6, nad1, cox2*) and three with ATA (*nad2, cox1, nad4*). The last one is classically found in the invertebrate mitochondrial genetic code, particularly in bivalves. Four *nad* genes start with NTG codons, corresponding to ATG (*nad1*), TTG (*nad3* and *nad5*) and GTG (*nad4l*). By contrast, PCGs are most often terminated by TAA (10 PCGs) rather than TAG (3 PCGs: *nad4, nad3, nad1*). These stop codons correspond to the only two possible combinations in the current invertebrate mitochondrial genetic code.

### Transfer and ribosomal RNA genes

The mitogenome of *Ma. balthica* is composed of 22 *tRNA* genes, which range in size from 61 to 69 bp. All of them fold into cloverleaf secondary structures with four arms, some of them presenting folding differences (Additional file 3). Four tRNA (*tRNA*^*Pro*^, *tRNA*^*Cys*^, *tRNA*^*Leu2*^ and *tRNA*^*Ile*^) have a small supplemental stem loop, *tRNA*^*Asn*^ has not terminal TΨC loop, *tRNA*^*Ser1*^ has the dihydrouracil (DHU) stem replaced by a big DHU loop and three discriminator nucleotides were detected instead of the single nucleotide usually presents at the 5' end. Finally, *tRNA*^*Ala*^, *tRNA*^*Leu1*^ and *tRNA*^*Val*^ do not possess a discriminator nucleotide.

*rrnS* and *rrnL* have respective lengths of 880 bp and 1,361 bp, and an AT content of 65.1% and 64.2%. Among specimens, average divergence is low (*rrnS*: 0 to 3%, *rrnL:* 0 to 5%). Overall, the same *tRNA* and *rRNA* features characterize all six mitogenomes of *Ma. balthica*.

### Non-coding regions: intergenic spacers and putative CR

Intergenic nucleotides represent 1,532 bp in total (*i.e.* 9.12% of the whole mitogenome), and are divided into 24 non-coding regions (NCRs, Table 2). The largest NCR is about 1,200 bp long (*i.e.* 7.14% of the whole mitogenome), is located between *rrnS* and *tRNA*^*Met*^ and is thought to contain the Control Region (CR). The putative CR could not be fully sequenced (either with MiSeq or Sanger sequencing, despite multiple attempts) due to its high AT content (estimated ~ 70%) and numerous tandem repeat motifs, but its size was estimated based on PCR products visualized by gel electrophoresis in strongly denaturing conditions. The other 23 NCRs ranged from 1 to 121 bp. Three overlapping gene regions were detected (*nad6* – *rrnL*: −32 bp, *tRNA*^*Gln*^ – *tRNA*^*Cys*^: −1 bp and *nad5* – *tRNA*^*Arg*^: −50 bp).

### Characterisation of a putative *atp8* gene

Local tblastx searches detected the full set of 13 mitochondrial PCGs characterized here among the 454 transcriptome sequences available for *Ma. balthica* [24], including an *atp8*, a gene long reported as rare in bivalves. This is the first record of an *atp8* gene in the Tellinidae. Up to now, this gene had not been highlighted in *Mo. iridescens* [26]. However, using the same data analysis approach as *Ma. balthica*, an *atp8* gene was also detected between *tRNA*^*Met*^ and *tRNA*^*Ser1*^ in publicly available *Mo. iridescens* sequences. Indeed, out of 84 mitogenomes of bivalves freely available on GenBank database, *atp8* gene had been detected in only 42 species.

In *Ma. balthica*, this short gene was detected between *tRNA*^*Met*^ and *tRNA*^*Ser1*^ and is separated from *atp6* by 1,970 bp. It encodes a 42 aa protein (Table 2) terminated by a complete stop codon (TAA). Conversely to other bivalves (Additional file 4), but as in *Mo. iridescens*, ATP8 protein starts with an isoleucine rather than a methionine. Despite an important length polymorphism in bivalve ATP8 (range of 33 to 109 aa length), 5/42 aa in *Ma. balthica* are shared among all investigated bivalves with an *atp8* (considering aa with > 75% conservation) and within the Tellinidae*, Mo. iridescens* and *Ma. balthica* have a 84% aa identity.

**Figure 2:**
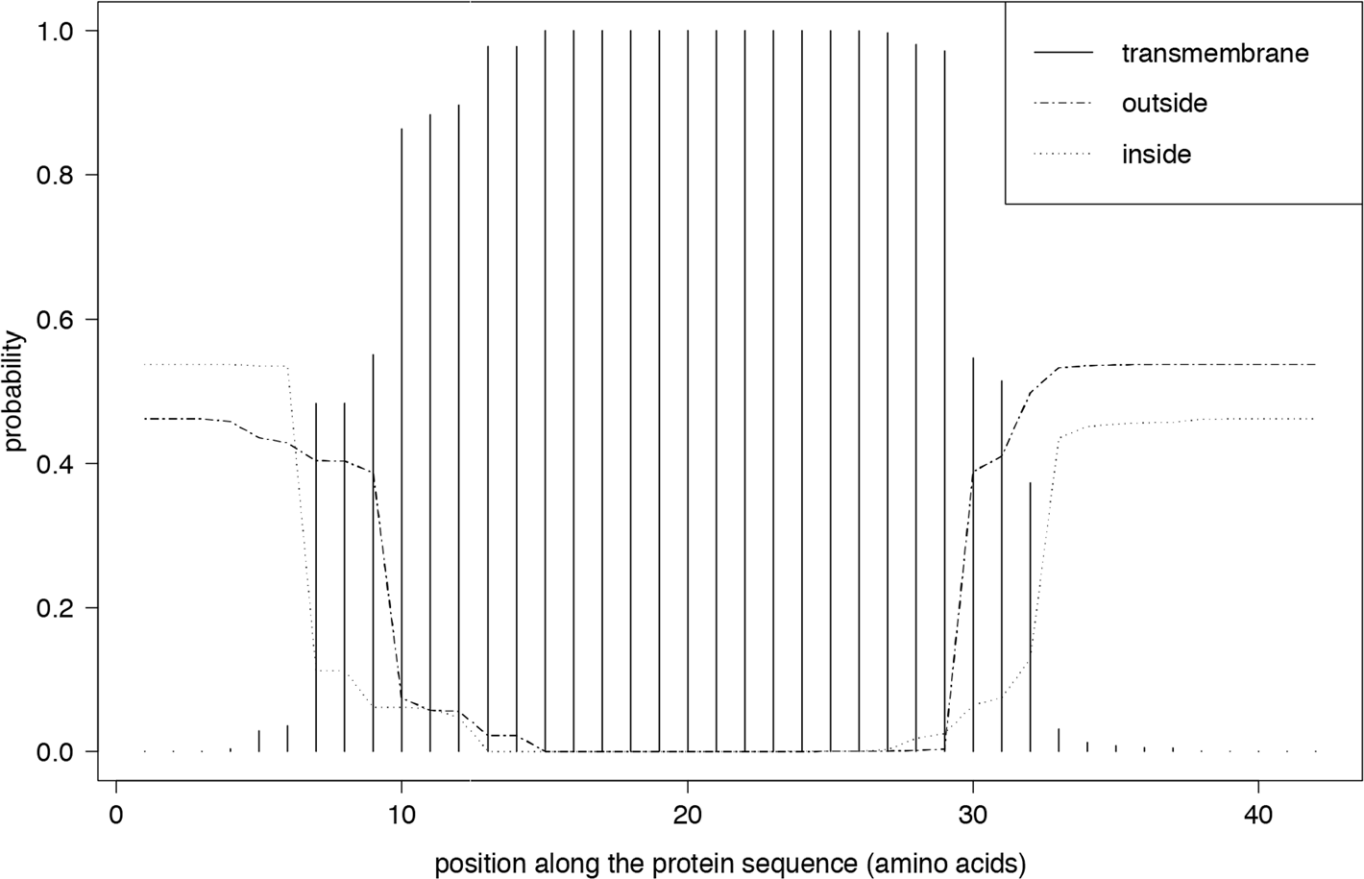
**Prediction of transmembrane helices in the** ***atp8*** **gene of** ***Ma. balthica*** **.**

Secondary structure is highly conserved. Indeed, *Ma. balthica* ATP8 hydrophobicity patterns are congruent with the typical transmembraneous protein pattern for this gene in bivalves (Figure 2 and Additional file 4). It is composed of a central hydrophobic part corresponding to a transmembraneous helix (aa 7 to 29) and two hydrophilic N-terminus (inter-membraneous space, aa 1 to 6) and C-terminus (matrix space, aa 30 to 42) extremities. These two domains are positively charged whereas the central part is encoded by negatively charged aa. TMHMM transmembraneous helix prediction of the ATP8 protein from *Ma. balthica* are congruent with these observations.

### PCGs: sequence divergence and polymorphism

PCG-specific haplotype network topologies (Additional file 5) differ in resolution (built based on data from six specimens, number of haplotypes detected varied from three to six) but present nearly identical evolutionary relationships. The *Ma. balthica balthica* (F17) and *Ma. balthica rubra* (A6, A10, M12, W4 and W20) lineages are the most divergent; within the *Ma. balthica rubra* clade (denoted "*intra-rubra*" hereafter), W20 is separated from W4 and other, more southern individuals.

**Figure 3:**
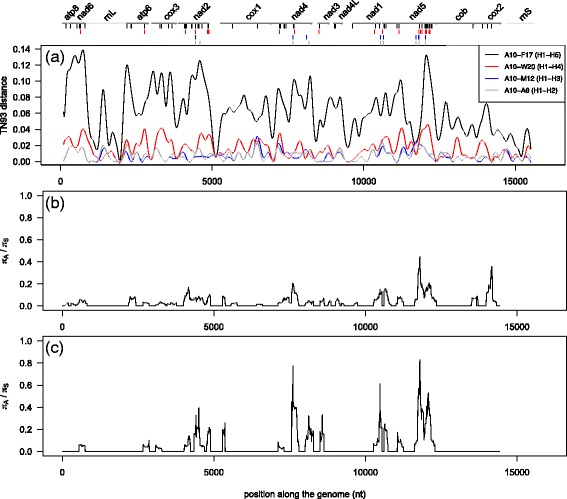
**Nucleotide divergence among specimens along the mitogenome of** ***Ma. balthica*** **.** Top: boundaries of protein-coding genes (black) and ribosomal genes (grey). Haplotypes H1-H5 represent *cox1* haplotypes (see Methods). Vertical bars represent the position of non-synonymous mutations for the four *cox1* haplotype comparisons, **(a)** divergence as measured by the Tamura and Nei [42] model of nucleotide substitution (TN93; substitution/site) and π_A_/π_S_ ratio for **(b)** all six mtDNA and **(c)** the *Ma. balthica rubra* lineage (n = 5 mtDNA).

Some regions (*rrnL*, five *tRNAs* between *nad2* and *cox1*, *rrnS*) are highly conserved and mutations are not evenly distributed along the 13 PCGs (Figure 3). The TN93 distance among the six mitogenomes ranged from 0.04 to 6.27% (median = 1.66, mean = 2.67) contrasting with 0.04 to 1.74% within *intra-rubra* (*i.e.* median = 0.7, mean = 0.92). The seven *nad* genes are highly mutated in comparison to the four *cytochrome* genes and two *atp* genes (Figure 3a). A10-F17 is the most divergent specimen pair (peak at 0.14 substitution/site at *nad6* and *nad5*; and 0.125 substitution/site at *nad2*) compared to other specimen pairs (max. peak at 0.05 substitution/site in *nad5* for the A10-W20 pair).

The ratio of intraspecific nucleotide diversity at non-synonymous (π_A_) and synonymous (π_S_) sites is lower when including both subspecies (Figure 3b) than within the *intra-rubra* group (Figure 3c). Overall, the *nad* gene group exhibits higher values of π_A_/π_S_ than the *atp* and *cytochrome* genes (Figures 3b,c and Table 3). Among *Ma. balthica* lineages, π_A_/π_S_ was most elevated in *nad2, nad4, nad1, nad5* and unexpectedly in *cox2* (Figure 3b; Table 3). The same pattern (except for *cox2*) was observed in the *intra-rubra* group (Figure 3c). Interestingly π_A_/π_S_ ratios for these four *nad* genes are slightly higher in the *intra-rubra* group than among *Ma. balthica* lineages (Table 3). Minimal π_A_/π_S_ ratio values were obtained in *atp6, 8*, *cox1, 3* and *cob* genes (Table 3).

**Table 3:** **Distribution of amino-acid (aa) changes on the 13 protein coding genes (PCGs) of** ***Macoma balthica***

**Lineage comparison level**	**PCG**	**Gene length (aa)**	**Number of aa change (% of total length)**	**Extramembraneous compartment**		**Intermembraneous compartment**		**Intramembraneous compartment**		**Nucleotide divergence** ^**1**^	**πA/πS ratio**
				**Length (aa)**	**Number of aa change**	**Length (aa)**	**Number of aa change**	**Length (aa)**	**Number of aa change**		
Among *M. balthica* (n = 6)	*atp8*	42	8 (19.1)	5	0	26	5	11	3	0 - 8.74 *(3.35 ± 1.64)*	0.03
	*nad6*	177	51 (28.8)	37	10	109	32	31	9	0 - 13.76 *(5.28 ± 2.25)*	0.027
	*atp6*	236	38 (16.1)	27	3	120	21	89	14	0 - 7.33 *(2.98 ± 1.24)*	0.031
	*cox3*	270	54 (20)	87	23	141	24	42	7	0.12 - 9.21 *(3.97 ± 1.58)*	0.017
	*nad2*	336	66 (19.6)	34	12	248	46	54	8	0.2 - 10.09 *(3.96 ± 1.71)*	0.065
	*cox1*	556	83 (14.9)	108	14	286	43	162	26	0.06 - 6.71 *(2.89 ± 1.13)*	0.004
	*nad4*	446	75 (16.8)	125	15	268	53	53	7	0 - 8.03 *(3.21 ± 1.33)*	0.039
	*nad3*	120	18 (15)	8	2	72	9	40	7	0 - 7.26 *(2.6 ± 1.15)*	0.027
	*nad4l*	96	18 (18.8)	34	7	56	10	6	1	0 - 8.38 *(3.29 ± 1.43)*	0.033
	*nad1*	307	43 (14)	46	8	203	29	58	6	0 - 6.24 *(2.52 ± 1.09)*	0.035
	*nad5*	597	78 (13.1)	114	16	361	52	122	10	0.06 - 7.32 *(3.31 ± 1.2)*	0.073
	*cob*	412	45 (10.9)	72	8	182	20	158	17	0 - 4.83 *(1.99 ± 0.81)*	0.008
	*cox2*	284	32 (11.3)	19	1	48	7	217	24	0 - 4.56 *(1.86 ± 0.8)*	0.043
Among *M. balthica rubra* (n = 5)	*atp8*	42	2 (4.8)	5	0	26	1	11	1	0 - 1.63 *(0.65 ± 0.34)*	0
	*nad6*	177	17 (9.6)	37	2	109	12	31	3	0 - 2.9 *(1.58 ± 0.5)*	0.017
	*atp6*	236	15 (6.4)	27	1	120	13	89	1	0 - 2.04 *(0.95 ± 0.29)*	0.02
	*cox3*	270	24 (8.9)	87	7	141	13	42	4	0.12 - 3.07 *(1.42 ± 0.53)*	0.011
	*nad2*	336	27 (8)	34	2	248	21	54	4	0.2 - 2.37 *(1.14 ± 0.4)*	0.084
	*cox1*	556	38 (6.8)	108	4	286	25	162	9	0.06 - 1.97 *(1.05 ± 0.3)*	0
	*nad4*	446	29 (6.5)	125	7	268	18	53	4	0 - 1.75 *(1.02 ± 0.25)*	0.064
	*nad3*	120	6 (5)	8	0	72	5	40	1	0 - 1.7 *(0.73 ± 0.29)*	0.073
	*nad4l*	96	6 (6.3)	34	3	56	3	6	0	0 - 1.4 *(0.91 ± 0.2)*	0
	*nad1*	307	15 (4.9)	46	1	203	10	58	4	0 - 1.43 *(0.72 ± 0.26)*	0.057
	*nad5*	597	50 (8.4)	114	11	361	30	122	9	0.06 - 2.64 *(1.4 ± 0.45)*	0.118
	*cob*	412	17 (4.1)	72	4	182	10	158	3	0 - 1.39 *(0.67 ± 0.22)*	0
	*cox2*	284	11 (3.9)	19	4	48	1	217	6	0 - 1.07 *(0.54 ± 0.17)*	0

^1^global divergence: min - max range (mean ± standard-error) calculated using the TN93 model of nucleotide substitutions, as for Figure 1.

The number of aa changes (*i.e.* synonymous and non-synonymous mutations) is given for each gene overall and for the extra-, inter- and intra-membranous organellar compartments, as delimited in Protter (Additional file 6). The distribution of aa changes across different organellar compartments provides a preliminary roadmap for searching for MNIs. The positions of aa sites bearing synonymous and non-synonymous mutations are mapped on the inferred protein secondary structures presented in Additional file 6.

Synonymous and non-synonymous mutations were mapped onto the PCG secondary structured inferred by Protter in order to evaluate the potential for MNIs (Additional file 6). While the sliding window analysis was run using pairs of individuals, the Protter analyses were run jointly on all samples. Mutation mapping among *Ma. balthica* lineages reveals that *nad6*, *cox3, nad2, atp8* and *nad4l* are the most mutated PCGs (number of aa changes ≥ 18.5% of total protein length), whereas only 11% of *cob* and *cox2* are mutated (Table 3). Other PCGs possess intermediate divergence levels, which varied from 13 to 16%. Within *intra-rubra* group, the most mutated genes are *nad6*, *cox3*, *nad5* and *nad3* (number of aa changes ≥ 8% of total protein length), and *atp8* was one of the most conserved genes, with *cox2* and *cob* (number of aa changes ≤ 5% of total protein length). On average *nad* genes have more aa bearing bp mutations than any other genes (5/7 *nad* genes have ≥ 15% of mutated sites among *Ma. balthica* and ≥ 6% within *intra-rubra* group; Table 3). The number of non-synonymous mutations was twice higher among *Ma. balthica* lineages than within the *intra-rubra* group (57 *vs* 30 non-synonymous mutations), with a maximum occurrence observed within the *nad2* and *nad5* genes (total of 16 and 24 respectively, Table 3). By contrast to *nad* genes, *atp6* and *atp8* genes present few non-synonymous mutations (total of 1 and 4 respectively, Table 3). Finally, the type and number of mutations were not influenced by the location of the mutation relative to the mitochondrial membrane (extra, inter or intramembranous positions; Fisher exact test, *P* > 0.05).

## Discussion

### General mitogenome features

Studies of metazoan mtDNA have revealed a large degree of variation in gene order that can be explained by multiple rearrangement events as inversions, transpositions, inverse transpositions (for details see [47]). In bivalves, on average, the number of mtDNA PCGs is 37 ± 3 [48], and genome architecture varies depending on the subclass. Members of Paleoheterodonta present the same gene order whereas Pteriomorpha and most importantly Heterodonta (a sub-class including *Macoma*) are widely rearranged (for a review see [26,49-51]).

The mitogenome of *Ma. balthica* (16.8 kbp) is among the smallest within the heterodonts (mean of 19 kbp, NCBI Organelle Genome Resources) ranging between *Acanthocardia tuberculata* (the smallest heterodont mitogenome with 16,104 bp [52]) and *Venerupis philippinarum* (the largest heterodont mitogenome with 22,676 bp [53]). The mitogenomes of *Ma. balthica* and *Mo. iridescens* [26] are similar in PCG length but present some differences in their start and stop codons. Genomic architecture is generally highly conserved within mollusc species. For example, the mitogenomes of two individuals of the clam *Meretrix lamarckii* have the same number and order of genes, slightly differed in length, and have identical amino-acid sequences [54]. Similarly, total genome length varied by < 1% among the six mitogenomes sequenced here, and genome organisation was identical.

### *atp8,* a dispensable gene in bivalves?

In metazoans, the number of genes is highly conserved (n = 37) and deviations from this number are rare. Among PCGs, the ATP synthase complex can be encoded by a maximum of three different genes corresponding to mitochondrial subunits 6, 8 and 9 (this latter subunit being present in most sponges [48]). However, in many bivalves, especially heterondonts, *atp8* is lacking, as in the Mactridae (NC_023384 and [50]), Arcticidae (NC_022709), Solenidae [55,56], Semelidae, Solecurtidae and Psammobiidae [26,57]. The first bivalve *atp8* was detected in [52] and was later found in the Cardiidae [49], Lucinidae (YP_003208152, YP_003208299) and Veneridae ([25,54,58,59] and YP_008854384). Here, the *atp8* gene is reported for the first time in the Tellinidae. Strongly divergent features of *atp8* among bivalves (lack of the MP** amino acid signature at the N-terminus, size length) could have previously hampered the annotation of this gene in the Tellinidae [60]. In other terms, this gene may evolve so fast in bivalves that it may be missed by homology analyses [60]. In mussels, Śmietanka et al. [61] highlighted that the number of nucleotide substitutions in PCGs is in average three times faster in the male mitochondrial lineage than in the female lineage, and varied differentially between respiratory complexes (from 2.6x for the first and fifth complexes to 8.5x for the third complex [61]). Strong divergence and fast evolution of this gene could also explain why it was not originally detected in *Mo. iridescens*.

Overall, gene length is highly variable among phyla (see [62]), raising the question of its role and dispensability in the functionality of the ATP synthase complex [48,63,64]. To date, the role of *atp8* stays unclear and little documented in metazoans. The N- and C-terminal domains of ATP8 seem to be involved in ATP synthase activity, particularly assembly and function of the F_O_ rotor [62] and was reported as an essential module in the stator stalk of yeast mtATP*ase* [63,64]. Stephens et al. [64] have demonstrated that the N- and C-terminal parts of yeast ATP8 were located in the intermembranous space and the mitochondrial matrix, respectively, while the rest of the protein was a transmembraneous, central hydrophobic domain. Our results in *Ma. balthica* are congruent with these observations, supporting the potential functionality of the ATP8 protein.

Some reviews [25,52] have attempted to make the link between ATP8 functionality and its genomic position relative to ATP6. Indeed, as mentioned by Boore [47], in mammals these two PCGs are "translated from a bicistronic transcript, with translation initiating alternatively at the 5′ end of the *mRNA* for *atp8* or at an internal start codon for *atp6*." Usually, these two genes are adjoined on the same strand, with overlapping reading frame [47]. Nonetheless, they can also be physically separated while both retaining functionality. Alternatively, *atp8* may be disjoint from *atp6* and non functional (for review see [62]). In *Ma. balthica*, *atp6* and *atp8* are separated by 1,970 bp but seemed to lead to functional proteins (at the least, both genes are expressed, as they were detected in our transcriptome database and presented the same PCG profile as other bivalves). This arrangement could be an "evolutionary stepping stone from the fully functional *atp6-atp8* coupling, via decoupled but complete genes" [52].

Yeast ATP8 is thought to interact with ATP synthase subunits b, f, 6 and is also able to cross-link with stator subunit d [64]. Moreover, assembly of subunits 8 and 6 into ATP synthase requires direct interactions between them [64]. Yeast F_O_-F_1_-ATP synthase requires five indispensable subunits (nuclear: b, d, f and mitochondrial: 6, 8) for stator stalk stabilization and loss of any one of these subunits results in a deep functional uncoupling between the F_1_ stator and the F_O_ rotor ([64] and references therein). This gene, if functional, may therefore coevolve with other ATP synthase subunits, and be involved in MNIs. Indeed, the sixth aa of the C-terminal domain of ATP8 is different in the *Ma. balthica balthica* and *Ma. balthica rubra* lineages, and multiple non-synonymous mutations were detected in the inter-membranous loops of ATP6. Furthermore, substitution of only one aa is sufficient to change protein stability [65]. This adaptive change could induce conformational modifications and impacted functionality role of PCGs.

### PCGs: polymorphism, divergence and selective pressure

A previous *F*_ST_ scan based on nuclear transcriptome data, between populations of *Ma. balthica* spanning the same two secondary contact zones as considered here, detected strong population structure at several genes involved in the ATP synthase and NADH deshydrogenase complexes [24]. These complexes both rely on the coevolution of multiple nuclear- and mitochondrial-encoded protein subunits, and could therefore suffer from MNIs. Nuclear and mitochondrial data converge toward positive selection pressures on the NADH dehydrogenase (Complex I of the OXPHO chain). The *nad* gene group is characterized by high polymorphism levels and high relative prevalence of replacement mutations compared to other PCGs. In particular, the *nad2* and *nad5* genes were rich in replacement mutations. Comparing the mitogenomes of the European and American eels (*Anguilla anguilla* and *A. rostrata*), Jacobsen et al. [16] found some evidence for positive selection on *atp6* (corroborating the results from [15]) and *nad5*. Inter-populational comparisons in the copepod *Tigriopus californicus* revealed that *nad3* was also among the most divergent PCGs, and displayed dN/dS ratios that were twice as elevated as other PCGs [3]. Similarly, Gibson et al. [13] have recently shown that hybrid breakdown in *Nasonia* parasitoid wasps is likely partly due to incompatibilities between nuclear- and mitochondrial-encoded OXPHO genes of Complex I. They identified the nuclear gene *Ndufa11*, coding for a subunit of the NADH dehydrogenase complex as strongly affected by transmission ratio distortion and marked by a prevalence of replacement mutations far greater than what was measured for other nuclear OXPHO genes [13,14]. Complex I ensures essential functions in the OXPHO chain, as it initiates electron transport system (ETS) and interacts with the OXPHO complexes II and III. Differential evolution of the mitochondrial- and nuclear-encoded NADH dehydrogenase subunits may therefore lead to coadaptation breakdown and possibly to MNIs. Further, because of its key role in the OXPHO chain, dysfunction of Complex I may severely affect all processes downstream of the OXPHO chain, and, ultimately, ATP production.

The mitochondrial *atp6* and *atp8* genes were not detected as outlier mitochondrial genes among the six individuals sequenced based on the number of non-synonymous mutations and π_A_/π_S_ ratios. This result contrasted with previous data, in which several nuclear-encoded ATP synthase subunits were highlighted as potentially being under positive selection [24]. Several factors could explain this result. First, the very small sample size used here (1–2 individuals sequenced per mitochondrial lineage) may have prevented us from detecting significant adaptive polymorphism. Indeed, the transcriptomic data suggested an asymmetric gene flow at the nuclear locus coding for the gamma subunit of ATP synthase, with a northern allele being apparently fixed in populations north of Brittany, and present in about 25% of reads in the population south of Brittany ([24] and unpublished data). It is therefore possible that the individuals selected here were not representative of the genetic diversity at mitochondrial *atp* genes. Second, a few adaptive mutations can have significant fitness impacts (*e.g.* Cytochrome *c* in *Tigriopus californicus* [66] and cMDH in *Lottia* [65]), and the relatively low prevalence of replacement mutations relative to neutral mutations may therefore not be indicative of the effect of protein evolution on fitness. Finally, genetic incompatibilities between ATP synthase subunits may arise solely between nuclear-encoded proteins.

We hope that future studies, focusing on experimental crosses and sequencing of additional ATP synthase subunits will help us determine whether these genes suffer from MNIs. Looking at the proportion of fixed and polymorphic mutations within and between *Ma. balthica* lineages using a larger sample size [67] will also help us investigate the adaptive value of protein divergence at OXPHO loci.

## Conclusions

The participation of MNIs to barriers limiting gene flow in the marine environment remains poorly known. In this contribution we characterized the mitochondrial genome of *Ma. balthica*, a high gene flow marine bivalve, in an effort to test whether mitochondrial genes involved in epistatic interactions with previously-detected nuclear outlier loci show stronger nucleotide and protein divergence than average. Our results suggest that genes coding for subunits of the NADH dehydrogenase protein complex could be involved in genetic incompatibilities. Further investigations will focus on larger intra- and inter-lineage sampling for mitochondrial and nuclear genes of the OXPHO chain to more fully characterize MNIs. Also, experimental crossings *Ma. balthica rubra* individuals sampled on each side of the Brittany hybrid zone will help understand the functional effects of PCG divergence.

### Availability of supporting data

Mitogenome DNA sequences of the six individuals have been submitted to GenBank (see Table 1 for Accession numbers) at www.ncbi.nlm.nih.gov. [68]. Raw Illumina reads were deposited on NCBI’s Sequence Read Archive (SRA; Project Accession Number SRP051152).

## Additional files

Additional file 1: Appendix S1. Detail of the protocol used in LR-PCR. Additional file 2: Table S1. List of primers used in LR-PCR. Additional file 3: Figure S1. Cloverleaf structures of the 22 *tRNA* genes in the reference mitogenome A10 of *Ma. balthica*. Nomenclature for portions of *tRNA* secondary structure is illustrated on *tRNA* ^*Phe*^. Additional file 4: Figure S2. Complete alignment of all bivalve *atp8* amino acid sequences available on GenBank (species – accession number of *atp8* (aa length)). Accession numbers of *atp8* sequences are not available for the two species of Tellinidae. For *Ma. balthica* and *Mo. iridescens* accession numbers of mitogenomes correspond to KM373200 and JN398362, respectively. Amino-acid hydrophobicity as described in [38] and references therein. Cytoplasmic side, transmembrane helix and mitochondrial matrix were defined following results for the subclass Heterodonta (transmembrane helix prediction in TMHMM). Additional file 5: Figure S3. Statistical parsimony haplotype networks for each PCG of *Ma. balthica*. The number of haplotypes present in each network is given in parentheses. Additional file 6: Figure S4. Mutation mapping for the 13 PCGs of *Ma. balthica*. A10 is taken as the reference and compared to other mitogenomes. Gray circles: synonymous changes in amino-acid (aa) among *Ma. balthica* lineages (*i.e.* all mitogenomes included); gray diamonds: synonymous changes in aa among *Ma. balthica rubra* lineages (*i.e.* excluding *Ma. balthica balthica* (F17)); black circles: non-synonymous changes in aa among *Ma. balthica* lineages; black diamonds: non-synonymous changes in aa among *Ma. balthica rubra* lineages.
