# Supplementary material for: Mitochondrial genomes of the Baltic clam Macoma balthica (Bivalvia: Tellinidae): setting the stage for studying mito-nuclear incompatibilities
Source: BMC Evol Biol. 2014 Dec 21;14:259. doi: 10.1186/s12862-014-0259-z (PMC4302422; doi:10.1186/s12862-014-0259-z)
Supplement: Additional file 1: Appendix S1. — Detail of the protocol used in LR-PCR. [file 12862_2014_259_MOESM1_ESM.docx]

**Mitochondrial genomes of the Baltic clam *Macoma balthica* (Bivalvia: Tellinidae): setting the stage for studying mito-nuclear incompatibilities**

A. Saunier, P. Garcia, V. Becquet, N. Marsaud, F. Escudié and E. Pante

**Additional file 1: Appendix S1.** Detail of the protocol used in LR-PCR.

The mitogenome was either amplified in two (using the COIF/CO3R and CO3F/COIR primer pairs) or three (using the COIF/16SR; 16SF/CO3R and CO3F/COIR primer pairs) fragments. Two different reactions mixtures, depending on the expected length of the PCR products, were used in a total volume of 50 μL. For short fragments (about 3 kbp, Additional file 2), reactions contained 1X PCR buffer, 1.5 mM MgCl_2_, 0.4 mM dNTPs, 0.2 μM of non-degenerated primer and 0.4 μM of degenerated CO3 primer, 2.5 U of Taq DNA polymerase (VWR, Radnor, PA, USA) and 50 to 100 ng of template DNA. For long fragments (16.5 and 13 kbp, Additional file 2) reactions contained 1X Long PCR Buffer with 1.5 mM MgCl_2_ (Fermentas Life Sciences, Hanover, MD, USA), 0.8 mM dNTPs, 0.2 μM of non-degenerated primer and 0.4 μM of degenerated CO3 primer, 2 U de Taq polymerase (Long PCR Enzyme Mix, Fermentas) and 100 to 200 ng of template DNA. LR-PCR were performed with a Labcycler gradient PCR machine (SensoQuest Biomedizinishe Elektronic GmbH, Göttingen, Germany) with initial denaturation and final extension steps of 1 and 3 min at 94°C for short and long fragments, respectively, and 10 min at 72°C. Other LR-PCR amplification conditions were optimized for each primer pair (Additional file 2).
