# Supplementary material for: Mitochondrial genomes of the Baltic clam Macoma balthica (Bivalvia: Tellinidae): setting the stage for studying mito-nuclear incompatibilities
Source: BMC Evol Biol. 2014 Dec 21;14:259. doi: 10.1186/s12862-014-0259-z (PMC4302422; doi:10.1186/s12862-014-0259-z)
Supplement: Additional file 2: Table S1. — List of primers used in LR-PCR. [file 12862_2014_259_MOESM2_ESM.docx]

**Mitochondrial genomes of the Baltic clam *Macoma balthica* (Bivalvia: Tellinidae): setting the stage for studying mito-nuclear incompatibilities**

A. Saunier, P. Garcia, V. Becquet, N. Marsaud, F. Escudié and E. Pante

**Additional file 2: Table S1.** List of primers used in LR-PCR. The “Product size” column contains the primer number given in the first column, followed by the approximate fragment length. The “Cycle program” column corresponds to number of PCR cycles (in brackets), temperature (°C): time (s) of the denaturation and annealing/extension phases. *primer pairs 1-6 and 1-4 are amplified using a two-step cycling protocol (annealing and extension coupled into one phase).

|  | **Primer name** | **Gene name** | **Sequence** | **Size (nt)** | **Tm (°C)** | **GC (%)** | **Product size (kbp)** | **Cycle program** | **Reference** |
| --- | --- | --- | --- | --- | --- | --- | --- | --- | --- |
| **1** | cox1-F_142169 | *cox1* | CTGCTTGGTTAGGTCATCCTGCCCCAGC | 28 | 71.0 | 60.7 | 1-6: 13 | [10]94:20,62:10/[25]94:20,62:15* | This study |
| **2** | cox1-R_108139 | *cox1* | GTAAAGTGTTCAACCTGTCCCAACCCCTTCCT | 32 | 69.5 | 50 | 2-3: 3 | [35]98:20,68:5 | This study |
| **3** | cox3-F_138168 | *cox3* | TAGGGTGTCAYACTTCTATTGTGGCYCGAGG | 31 | 69.5 | 51.6 |  |  | This study |
| **4** | cox3-R_138168 | *cox3* | CCTCGRGCCACAATAGAAGTRTGACACCCTA | 31 | 69.5 | 51.6 | 1-4: 16.5 | [10]94:20,68:12/[20]94:20,68:17* | This study |
| **5** | rnl_16Sar-5'F | *rnL* | CGCCTGTTTATCAAAAACAT | 20 | 51.2 | 35 | 4-5: 3 | [35]98:20,50:5 | [28] |
