# Supplementary material for: Mitochondrial genomes of the Baltic clam Macoma balthica (Bivalvia: Tellinidae): setting the stage for studying mito-nuclear incompatibilities
Source: BMC Evol Biol. 2014 Dec 21;14:259. doi: 10.1186/s12862-014-0259-z (PMC4302422; doi:10.1186/s12862-014-0259-z)
Supplement: Additional file 3: Figure S1. — Cloverleaf structures of the 22 tRNA genes in the reference mitogenome A10 of Ma. balthica. Nomenclature for portions of tRNA secondary structure is illustrated on tRNA Phe. [file 12862_2014_259_MOESM3_ESM.pdf]

Additional file 3: Figure S1. Cloverleaf structures of the 22 *tRNA* genes in the reference mitogenome A10 of *Ma. balthica*. Nomenclature for portions of *tRNA* secondary

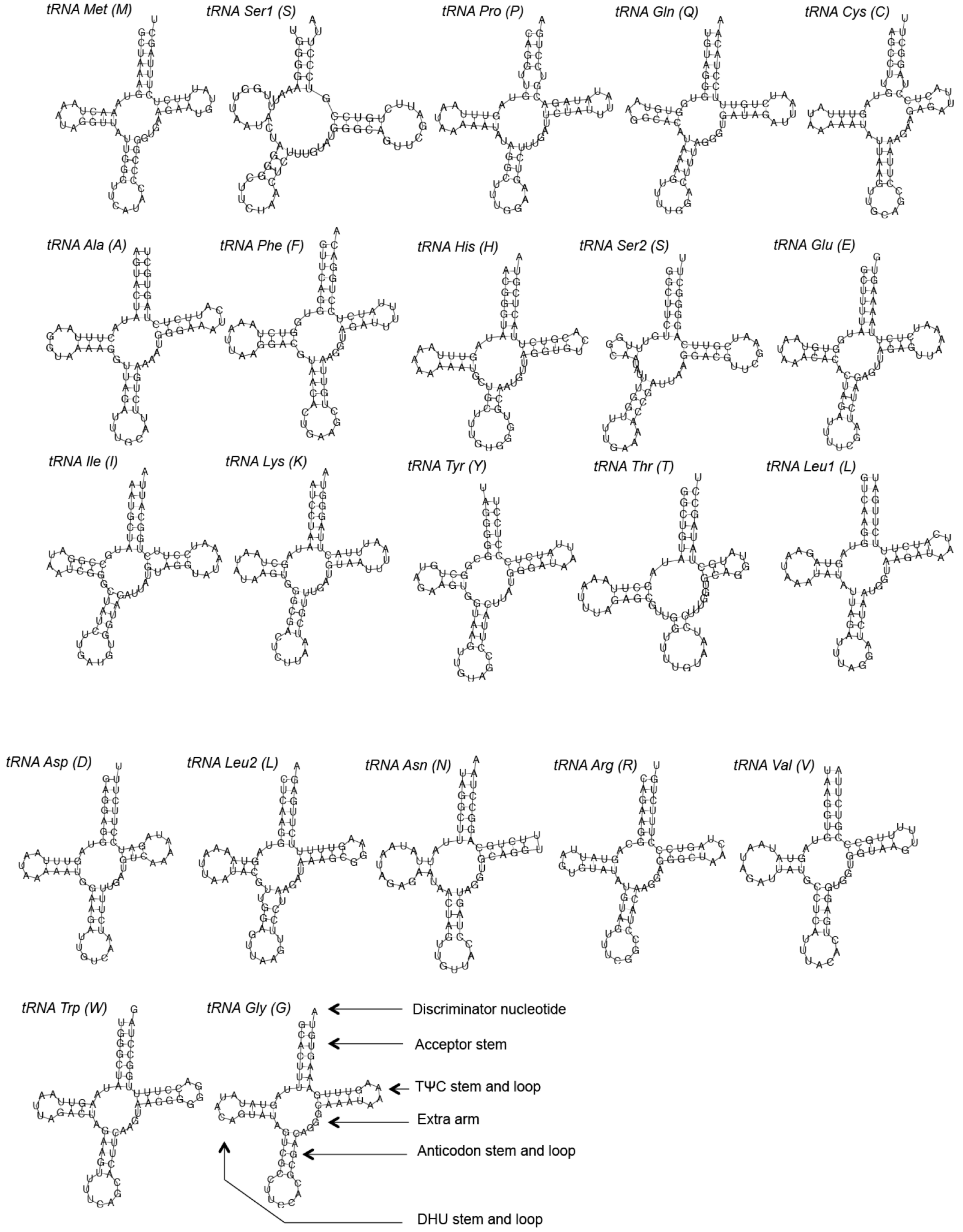

structure is illustrated on $tRNA^{Phe}$.
