# Supplementary material for: Mitochondrial genomes of the Baltic clam Macoma balthica (Bivalvia: Tellinidae): setting the stage for studying mito-nuclear incompatibilities
Source: BMC Evol Biol. 2014 Dec 21;14:259. doi: 10.1186/s12862-014-0259-z (PMC4302422; doi:10.1186/s12862-014-0259-z)

Additional file 4: Figure S2. Complete alignment of all bivalve *atp8* amino acid sequences available on GenBank (species – accession number of *atp8* (aa length)). Accession numbers of *atp8* sequences are not available for the two species of Tellinidae. For *Ma balthica* and *Mo iridescens* accession number of mitogenomes corresponds [KM373200] and [JN398362], respectively. Amino-acid hydrophobicity as described in [38] and references therein. Cytoplasmic side, transmembrane helix and mitochondrial matrix were defined following results for the subclass Heterodonta (transmembrane helix prediction in TMHMM).

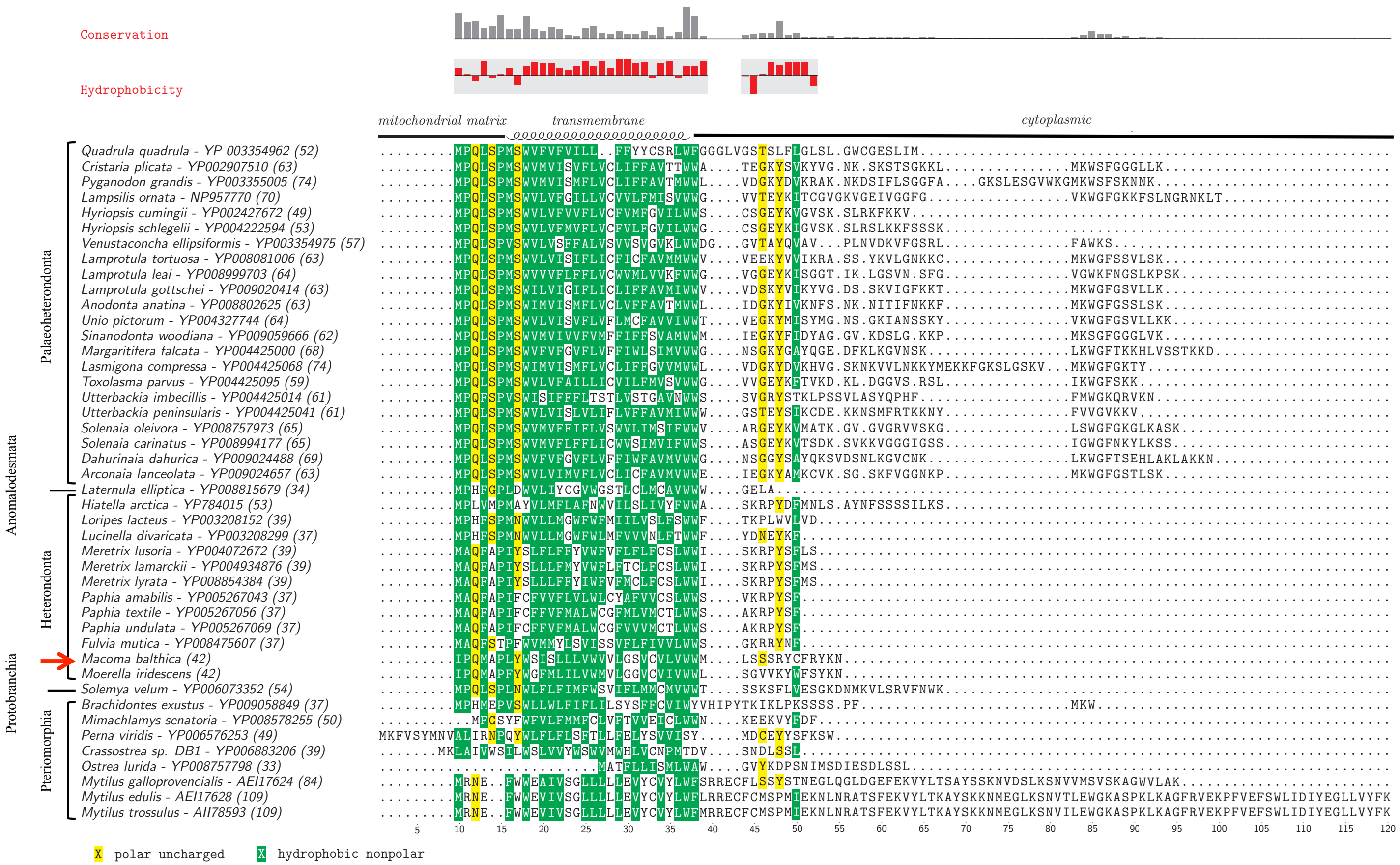

Supplement: Additional file 4: Figure S2. — Complete alignment of all bivalve atp8 amino acid sequences available on GenBank (species – accession number of atp8 (aa length)). Accession numbers of atp8 sequences are not available for the two species of Tellinidae. For Ma. balthica and Mo. iridescens accession numbers of mitogenomes correspond to KM373200 and JN398362, respectively. Amino-acid hydrophobicity as described in [38] and references therein. Cytoplasmic side, transmembrane helix and mitochondrial matrix were defined following results for the subclass Heterodonta (transmembrane helix prediction in TMHMM). [file 12862_2014_259_MOESM4_ESM.pdf]
