# Supplementary material for: Mitochondrial genomes of the Baltic clam Macoma balthica (Bivalvia: Tellinidae): setting the stage for studying mito-nuclear incompatibilities
Source: BMC Evol Biol. 2014 Dec 21;14:259. doi: 10.1186/s12862-014-0259-z (PMC4302422; doi:10.1186/s12862-014-0259-z)

Additional file 5: Figure S3. Statistical parsimony haplotype networks for each PCG of *Ma. balthica*. The number of haplotypes present in each network is given in parentheses.

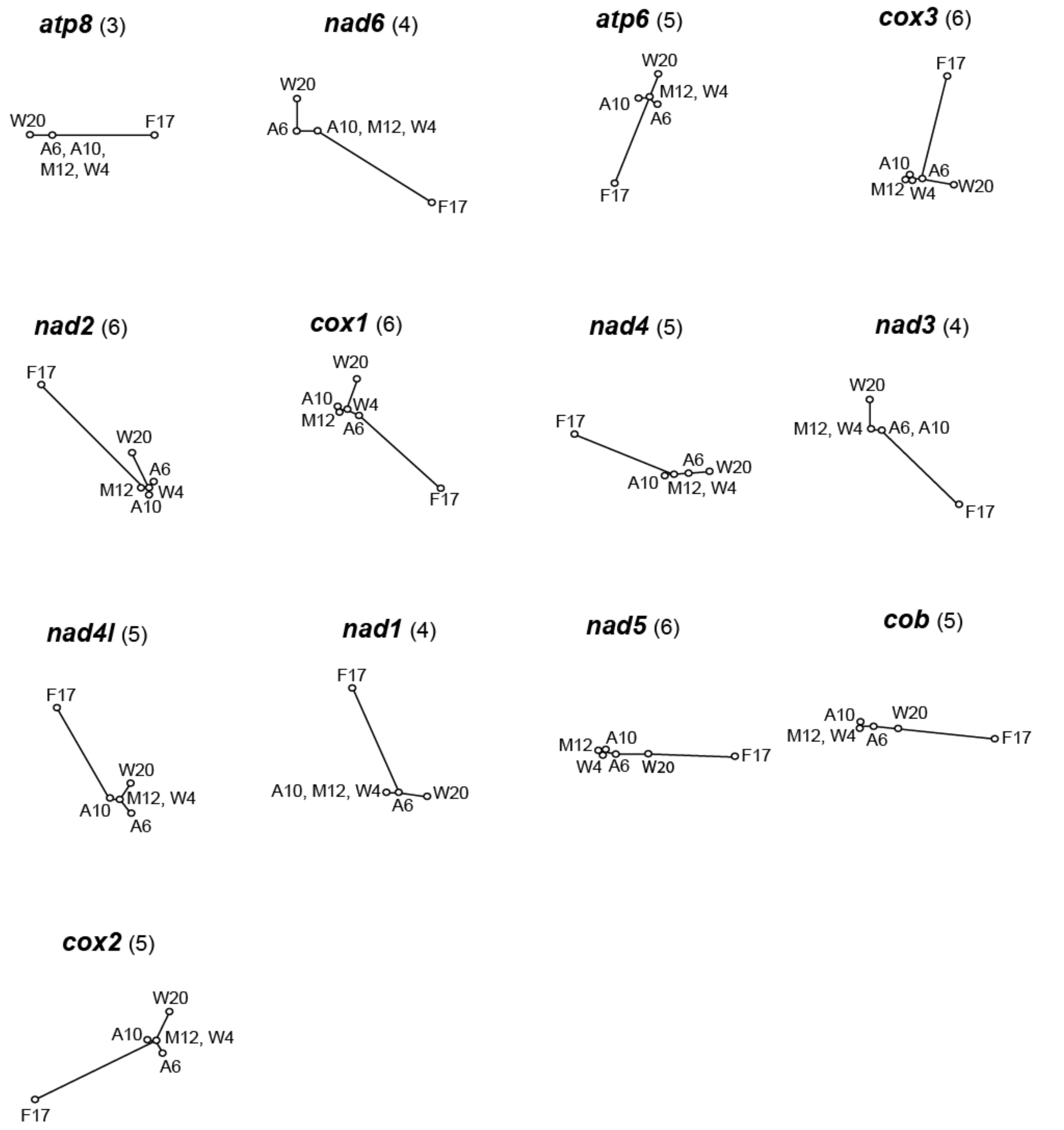

Supplement: Additional file 5: Figure S3. — Statistical parsimony haplotype networks for each PCG of Ma. balthica. The number of haplotypes present in each network is given in parentheses. [file 12862_2014_259_MOESM5_ESM.pdf]
