# Supplementary material for: Mitochondrial genomes of the Baltic clam Macoma balthica (Bivalvia: Tellinidae): setting the stage for studying mito-nuclear incompatibilities
Source: BMC Evol Biol. 2014 Dec 21;14:259. doi: 10.1186/s12862-014-0259-z (PMC4302422; doi:10.1186/s12862-014-0259-z)

Additional file 6: Figure S4. Mutation mapping for the 13 PCGs of *Ma. balthica*. A10 is taken as the reference and compared to other mitogenomes. Gray circles: synonymous changes in amino-acid (aa) among *M. balthica* lineages (*i.e.* all mitogenomes included); gray diamonds: synonymous changes in aa among *M. balthica rubra* lineages (*i.e.* excluding *M. balthica balthica* (F17)); black circles: non-synonymous changes in aa among *M. balthica* lineages; black diamonds: non-synonymous changes in aa among *M. balthica rubra* lineages.

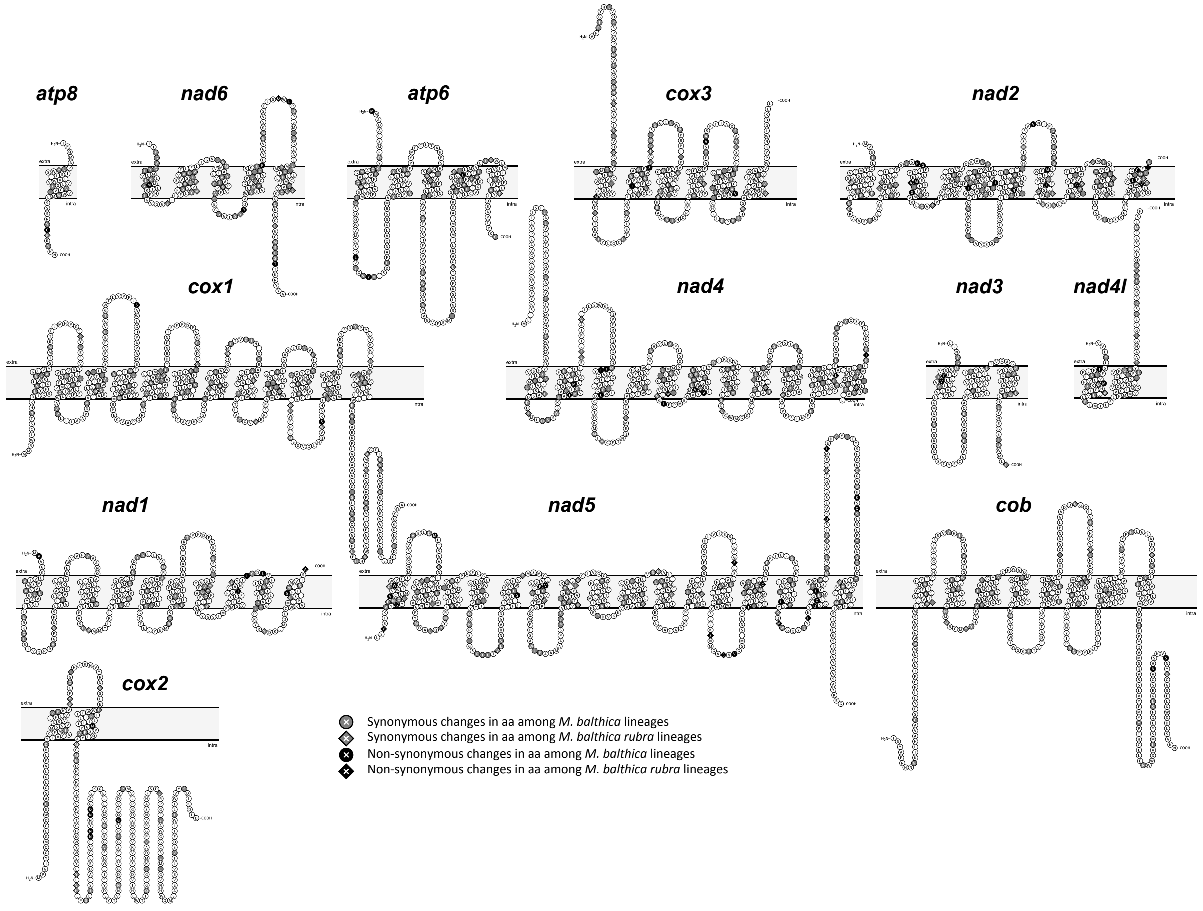

Supplement: Additional file 6: Figure S4. — Mutation mapping for the 13 PCGs of Ma. balthica. A10 is taken as the reference and compared to other mitogenomes. Gray circles: synonymous changes in amino-acid (aa) among Ma. balthica lineages (i.e. all mitogenomes included); gray diamonds: synonymous changes in aa among Ma. balthica rubra lineages (i.e. excluding Ma. balthica balthica (F17)); black circles: non-synonymous changes in aa among Ma. balthica lineages; black diamonds: non-synonymous changes in aa among Ma. balthica rubra lineages. [file 12862_2014_259_MOESM6_ESM.pdf]
